# Plant-Based Emulsions as Dairy Cream Alternatives: Comparison of Viscoelastic Properties and Colloidal Stability of Various Model Products

**DOI:** 10.3390/foods13081225

**Published:** 2024-04-17

**Authors:** Barbora Lapčíková, Lubomír Lapčík, Tomáš Valenta, Marie Chvatíková

**Affiliations:** 1 Department of Physical Chemistry, Faculty of Science, Palacky University Olomouc, 17. Listopadu 12, CZ-771 46 Olomouc, Czech Republic; barbora.lapcikova@upol.cz; 2 Department of Food Technology, Faculty of Technology, Tomas Bata University in Zlín, Nám. T. G. Masaryka 5555, CZ-760 01 Zlín, Czech Republic; tvalenta@utb.cz (T.V.); marie.chvatikova@gmail.com (M.C.)

**Keywords:** cream products, o/w emulsions, recipe formulation, emulsifier, rheological behaviour, viscoelastic moduli, colour parameters

## Abstract

In the context of growing interest in plant-based food products for their potential health benefits and sustainability, this study investigates the effect of mono- and diglycerides of fatty acids application on physico-chemical properties of various plant-based cream products, compared to lecithin application in rice cream. Rheological and textural parameters, colour profile, and colloidal stability were analysed. The application of mono- and diglycerides modified the creams’ viscoelastic behaviour, showing a decrease in viscoelasticity across the samples; although in oat–coconut cream resulted in a higher viscoelasticity, indicating the formation of a gel-like structure. Rice cream with lecithin emulsifier showed lower viscoelastic properties characterised by higher phase angle (*tan δ*). All samples behaved as pseudoplastic materials (with a flow behaviour index *n* < 1). For coconut and almond creams, the consistency coefficient increased and flow behaviour index decreased after emulsifier application. Interestingly, the emulsifier addition did not significantly affect the cream’s colour profile, characterised by yellow hue angle (h*) as a dominant spectral component. The colloidal stability, indicated by a stability index (*SI*), was determined as well.

## 1. Introduction

Plant-based creams are considered oil/water (o/w) emulsions that contain valuable nutrients, aromas, and flavours extracted by soaking and blending food ingredients [1]. In recent years, plant-based emulsions have attracted researchers’ attention due to their advantageous properties. Proteins, cellulose, starch, and polysaccharides have been used for particle stabilization, particularly in whipped creams, to create the stable and healthy emulsions where hydrogenated oils are replaced by liquid vegetable oils to increase the proportion of unsaturated fatty acids [2,3,4,5,6]. Rheological and texture profiles of plant-based emulsions play a crucial role in creams’ stability and food applications [7]. As an example, texture-modified foods for the elderly can be highlighted, which include a group of products, especially the categories from “thin drinks” to “pureed” with dynamic viscosity around 50 to 1750 mPa·s, respectively [8,9]. Rheological properties, which include viscosity, elasticity, and plasticity, are influenced by the interaction between the dispersed particles and the continuous phase of the emulsions. The viscoelastic behaviour of the products, characterised by their ability to exhibit both viscous (liquid-like) and elastic (solid-like) nature, is particularly important. It affects the creams’ spreadability, mouthfeel, and shelf-life stability [10]. The flow behaviour of the creams is another key property. Understanding these rheological attributes is essential for the formulation and processing of plant-based creams, as it allows for the prediction and control of their performance in various applications [7,10,11].

Nowadays, there is a significant interest in finding food alternatives with the aim of producing plant-based products as replacements for animal-based (dairy) creams, spreads, and emulsified sauces in order to improve human health and reduce food waste. Cream emulsions can be used as alternatives to classic cream in the preparation of soups, sauces, dips, porridges, and drinks. Coconut cream, in particular, is used in Thai dishes. Hence, it is important to enhance the physico-chemical properties of the creams so that they can be developed into convenient and sustainable products that are acceptable to a wide range of consumers. In this regard, research conducted in recent years has focused on specific aspects of the formation, composition, and properties of these creams. More recently, efforts have been made to design plant-based creams that simulate functional attributes of milk products [12,13].

Plant-based creams are gel-like liquids containing various substances and ingredients such as carbohydrates, proteins, fats, salts, insoluble compounds, hydrocolloids, etc. These dispersed substances interact with water to reduce water activity and increase product viscosity. Hydrocolloids contribute to these phenomena by reducing the tendency of dispersed particles to collapse. Nutritionally modified plant-based creams can be formulated to become health-specific food products suitable for individuals managing conditions such as heart disease, cancer, type II diabetes, and others [14,15,16].

The plant-based creams have the potential to act as a viable source of proteins originating from plant materials. Various cereals, including rice, oat, wheat, barley, etc., as well as legumes, nuts, and oilseeds, may be processed to prepare cream dispersions and matrices rich in protein content. In food formulations, plant-based proteins play an important functional role, acting as gelling, thickening, and foaming agents. These proteins are also capable of stabilizing emulsions [17,18,19,20]. Plant-based creams, due to their advantageous oil/fat profiles, can also be considered a suitable and healthy source of edible fats. These creams can serve as an effective substitute for unhealthy fatty diet [21]. Composed of liquid vegetable oils rich in unsaturated fatty acids, these substitutes can replace highly saturated animal fats and hydrogenated vegetable oils, thereby optimizing the fatty acid ratio in the food products. Besides this, plant-based foods are rich in bioactive compounds, including vitamin C, vitamin E, beta carotene, polyphenols, and phenolic acids. These compounds in the creams can contribute to antioxidant defence, lower inflammation, and promote the structural support of human skin [22].

However, some factors can modify the properties of plant-based creams in an undesirable way. These factors include time- and material-dependent changes in colloidal stability, the ratio of oil-to-water phases, water activity, fat reduction, and others [8,9]. These modifications may pose a great challenge. Commercial milk alternatives based on plant-based sources are sometimes poorly accepted by consumers due to the differences in quality, organoleptic characteristics, and in functional attributes, such as stability, creaming, undercooking, etc. [23]. This all necessitates creams’ standard preparation procedures, along with appropriate handling and storage.

Plant-based creams represent good candidates for scientific research and practical industrial applications. Their processing may be a vital topic for many studies regarding the production of healthy foods and diets for consumers with diabetes, lactose intolerance, and other diseases. Varying ingredient composition and preparation procedures can yield diverse products with various properties. The novelty of this study lies in the innovative approach to understanding the effect of applied mono- and diglycerides of fatty acids on the physico-chemical properties of plant-based creams. It provides new insights into how these emulsifiers alter the profiles of various cream emulsions, offering valuable information for the formulation and processing of food products. Specifically, the aim of this investigation was to compare the viscoelasticity and flow behaviour of different model cream types in view of the mono- and diglycerides application. The primary focus of this paper was to determine the rheological and textural patterns of the creams in relation to their composition and colloidal stability. The colour profiles of model products, characterised by lightness, chromatographic coordinates, hue angle, and chroma saturation, were evaluated as well. This study, therefore, represents an advancement in the field of potential plant-based food products development.

## 2. Materials and Methods

### 2.1. Plant-Based Creams’ Recipe Ingredients

Food ingredients were purchased from certified companies and used in the recipe formulations. The following ingredients were utilised: whole almonds (trademark: ARO; country of origin: USA; supplier: K-servis Praha a.s., Nučice-Rudná u Prahy, Czechia), organic oatmeal (trademark: Probio; manufacturer: Pro-Bio, s.r.o., Staré Město pod Sněžníkem, Czechia), round-grain husked rice (trademark: Gold plus Classic, s.r.o.; supplier: Gold-plus Company, s.r.o., Bratislava, Slovakia), grated coconut (trademark: ARO; supplier: K-servis Praha a.s., Nučice-Rudná u Prahy, Czechia), fine rice flour with an average particle radius of 67.4 μm (manufacturer: Adveni medical, s.r.o., Mělčany, Czechia), sunflower oil (trademark: ARO; manufacturer: MCC Trading International GmbH, Düsseldorf, Nordrhein-Westfalen, Germany), guar gum (product type: G4129-250G; producer: Sigma-Aldrich, Co., St. Louis, MO, USA), natural soy lecithin E322 (manufacturer: Mogador s.r.o., Otrokovice, Czechia), vacuum salt with iodine (manufacturer: K + S Czech Republic a.s., Prague, Czechia), and mono- and diglycerides of fatty acids E471 (glyceryl monostearate and glyceryl distearate; manufacturer: Jaroslav Kostera, J. K. Food s.r.o., Větřkovice, Czechia).

Mono- and diglycerides of fatty acids, which are part of common fats, can be used as emulsifier and polishing agent for fresh fruit and vegetables according to EU legislation (EU Commission Regulation 2019/801) [24]. Their application helps to protect the nutritional quality of the products and extend shelf life. In this study, the mono- and diglycerides were utilised as an emulsifier and stabiliser on a lipophilic basis in plant-based emulsions. Guar gum, a hydrocolloid stabiliser of a macromolecular nature, was hydrated in the aqueous phase, providing long-term physical stability to the creams. The stabilization of cream emulsions was achieved by restricting the mobility of dispersed phase droplets due to the increased viscosity and viscoelasticity of the continuous phase [12]. Tap drinking water with a conductivity of 25 mS/m was used for the preparation of all creams. The quality of the drinking water complied with the EC Directive 98/83/EC on the quality of water intended for human consumption [25].

### 2.2. Plant-Based Cream Preparation

Plant-based creams were prepared by blending food ingredients to prepare emulsions of the desired composition and consistency. Rice cream was prepared in a Thermomix TM 6 (Vorwerk SE & Co. KG, Wuppertal, Germany), where the rice was first cooked and then mixed at 2000 RPM with the ingredients listed in Table 1. As a typical emulsifier applied in rice, lecithin was used in the rice cream formulation to compare with other creams’ model products, emulsified with mono- and diglycerides of fatty acids. The almond, coconut, oat, and oat–coconut creams were prepared from basic ingredients (almonds, coconut, oatmeal, and oatmeal with coconut at a 1:1 (*w*/*w*) ratio, respectively) that had been soaked overnight and subsequently drained. The ingredients were then mixed using a Philips Viva Collection blender 600 W HR 2170 (Koninklijke Philips N.V., Amsterdam, North Holland, The Netherlands) at 11,500 RPM for 10 min. The amount of prepared samples was 500–600 mL (900 mL for rice cream), dependent on the bulk density of basic ingredients used. At this stage, sunflower oil and salt were added to the mixture, which was blended to a smooth consistency. The ratio of oil-to-water (o/w) in the cream emulsions was 0.1 vol.%. Guar gum, rice flour, and emulsifier (for emulsified creams) were gradually added to the mixture at the lowest rotational speed (40 RPM). Cream formulations were optimised based on the composition of the input raw materials in various product types; the optimised recipes are summarised in Table 1. The content of additives in the creams was governed by the Directive 95/2/EC of the European Parliament and Council of 20 February 1995 on “Food additives other than colours and sweeteners”. According to this directive, food additives in follow-on nutrition for healthy infants have a maximum limit that can be added to the products: 1 g/L of lecithin, 4 g/L of mono- and diglycerides of fatty acids, and 1 g/L of guar gum [26].

After the preparation, the cream products were poured into the plastic cups and cooled at a laboratory temperature of 24 ± 1 °C, then the creams were stored in a refrigerator at a temperature of 4 ± 1 °C and used for measurements taken within 24 h.

### 2.3. Rheological Analysis of Plant-Based Creams

The rheological analysis of plant-based creams was realised by rheometer Malvern Kinexus PRO (Malvern Panalytical Ltd., Malvern, UK). To determine flow behaviour of the creams of liquid consistency (MS), cylinder–cylinder geometry was used (sample volume: 17.1 mL; gap size: 9.15 mm). For creams of semi-solid or solid consistency, plate–plate geometry with a gap size of 2 mm was used [27]. The shear viscosity *η* as a ratio of shear stress to shear rate was measured in the shear rate range of 0.1–100 s^−1^ at a temperature of 25.00 ± 0.01 °C. To evaluate flow parameters of the samples, Ostwald–de Waele (Power law) and Herschel–Bulkley models were applied to fit the viscometric data. Ostwald–de Waele model was calculated as follows:

(1) $$\tau =k\cdot {\dot{\gamma }}^{n}$$

where *τ* is the shear stress (Pa), *k* is the consistency coefficient (Pa·s^n^), *n* is the flow behaviour index (dimensionless), and $\dot{\gamma }$ is the shear rate (s^−1^) [27].

The Herschel–Bulkley model represents the extension of the Ostwald–de Waele relationship. This model was used to describe the flow above a yield point. The Herschel–Bulkley model involves the yield stress *τ*_0_ (Pa) in the following form:

(2) $$\tau =\tau_{0}+k\cdot {\dot{\gamma }}^{n}$$

In some cases, Herschel–Bulkley modelling of flow curves provided negative yield stress which is physically meaningless. For that reason, to optimise the *τ*_0_ data of extremely low values extrapolated by the non-linear regression fit, the corresponding yield stress was assumed to be zero [27].

To determine viscoelastic behaviour of the creams, plate–plate geometry with a gap size of 2 mm (40 mm plate diameter) was used. A strain of 0.1% was applied to conduct an oscillation frequency sweep in the material equilibrium state, i.e., in linear viscoelastic region [28]. The measurement was performed to evaluate the change in elastic storage modulus (*G*′) and viscous loss modulus (*G*″) per frequency rate 0.1–20 Hz at a temperature of 25.00 ± 0.01 °C. Based on the viscoelastic moduli data, the phase (loss) angle *tan δ* as a ratio of *G*″/*G*′ was determined. Software *rSpace* version 1.50 (Malvern Panalytical Ltd., Grovewood Rd, Worcestershire, UK) was employed to evaluate the results.

### 2.4. Texture Analysis of Plant-Based Creams

The extural properties of plant-based creams were evaluated using a texture analyser TA.XTplus (Stable Micro Systems Ltd., Godalming, UK) equipped with TTC spreadability rig (product code HDP/SR), a 90° cone probe, and a transparent perspex cone-shaped sample holder. The analysis was performed by the 2 mm penetration of the cone probe into the sample (using a trigger force of 5 g and a deformation rate of 1 mm/s) at a temperature of 5 ± 1 °C. The sample was forced to flow outward at 45° between the cone surfaces during the test, indicating the degree to which the cream can be spread. The withdrawal of the cone probe from the sample provided information about the cream adhesive characteristics.

Samples were analysed for their spreadability, firmness, stickiness, and adhesiveness. These textural parameters were calculated based on the force/time curves. Firmness and stickiness were determined by the peak positive force and the peak negative force (N), respectively. The positive area of the force/time curve corresponded to the cream spreadability (N·s) [29]. The adhesiveness was determined as the absolute value of the negative force area, representing the attractive forces between the cream surface and the probe (N·s) [30].

Creams of relevant consistency, i.e., KS, KSE, OKSE, and RSL, were fit in order to be analysed. Other cream types in the study were not applicable to the texture analysis.

### 2.5. Colour Analysis of Plant-Based Creams

The colour analysis of plant-based creams was performed on Spectrophotometer UltraScan VIS (Hunter Associates Laboratory, Inc., Reston, VA, USA). Measurements were carried out using an Illuminant D65/10 (standard daylight with 10° angle). Creams were analysed in a 20 mm glass cuvette using measuring modes based on their surface nature. Creams showing a specular reflectance (MS, MSE, KSE, OS, OSE, OKS, OKSE, and RSL) were measured in RSIN (reflectance specular-included) mode, and the cream without a specular reflectance (KS) in RSEX (reflectance specular-excluded) mode. Both for RSIN and RSEX modes, the Diffusive 8 Instrument Standard was used as a reference. In accordance with the recommendation regarding the effect of background colour [31], the thickness of measured plant-based creams (20 mm) was sufficiently high to obtain relevant values of colour coordinates.

The chroma meter was adjusted to the CIE L*a*b* colour space to determine the lightness (luminosity) L* (0 = black; 100 = white), and chromaticity coordinates a* (from the greenness (−) to redness (+)), and b* (from the blueness (−) to yellowness (+)) [31,32,33]. Three reads were taken for each sample measured in three replicates. Due to the fact that the combination of a* and b* provides a more precise and characteristic indication of sample colour [33,34,35], hue angle h*, and colour saturation (chroma) C* were calculated from the following formulas:

(3) h*=arctan ⁡b*/a*

(4) $$C^{*}={\left[{a^{*}}^{2}+{b^{*}}^{2}\right]}^{0.5}$$

Whiteness and yellowness indices of the creams were evaluated as well. The whiteness index *WI* was calculated as follows [34]:

(5) $$WI={100-\left[{\left(100-L^{*}\right)}^{2}+a^{*2}+b^{*2}\right]}^{0.5}$$

The yellowness index *YI* was determined using the following formula [34]:

(6) $$YI=142.86\cdot \left(b^{*}/L^{*}\right)$$

### 2.6. Colloidal Stability of Plant-Based Creams

The stability of the creams was determined by centrifugation process using the centrifuge Hettich EBA 21 (Andreas Hettich GmbH & Co. KG, Tuttlingen, Baden-Württemberg, Germany). A total of 30 mL of the sample was centrifuged in conic polyethylene tubes at 6000 RPM for 20 min at laboratory temperature (25 ± 1) °C. Time-dependent phase separation was simulated by the centrifugation process, and the phase stability of the creams was evaluated by the stability index (*SI*) [36]. *SI* was calculated by Equation (7):

(7) $$SI\;\left(\%\right)=\left(1-V_{2}/V_{1}\right)\cdot 100$$

where *V*_2_ is total volume of the cream and *V*_1_ is the volume of oil phase (supernatant) after the centrifugation.

### 2.7. Statistical Analysis

Data with a normal distribution were evaluated using analysis of variance (ANOVA test) with a two-way experimental design. Two factors (model cream type, application of emulsifier) were tested to verify the effect of the factors on the parameters measured. For rheological analysis, the variables involved flow parameters, as described in Section 2.3. Analogously, relevant textural and colour parameters were involved in texture and colour statistics, respectively. For colloidal stability evaluation, the stability index (*SI*) of the creams was assessed. Differences in the mean values among statistical groups were tested at a significance level of *α* ≤ 0.05. Tukey’s test was applied for multiple comparisons of sample mean responses to the treatment groups. The statistical software SigmaStat version 2.03 (Systat Software, Inc., San Jose, CA, USA) was used for data testing. The creams were measured in three replicates; each replicate was tested three times.

## 3. Results and Discussion

### 3.1. Rheological Analysis Plant-Based Creams

Representative flow curves of plant-based emulsions showed shear-thinning behaviour (*n* < 1), as viscosity highly decreased when increasing shear rate. Flow parameters were evaluated by the Ostwald–de Waele and Herschel–Bulkley rheological models. The aforementioned models were fitted to the measured data from the flow curves with varying correlation coefficients, reflecting the statistical tightness of the data (the rheological data are presented in Table S1, Supplementary Materials). The results of the analysis are given in Table 2, characterised by the flow parameters of yield stress (*τ*_0_), consistency coefficient (*k*), flow behaviour index (*n*), and correlation coefficients (*R*^2^). It was found that the Herschel–Bulkley model was the most fitting one, with a *R*^2^ ≥ 0.99. Due to the fact that some samples showed a yield stress, it was not possible to generalise the flow behaviour of all measured samples. When the samples exhibited zero yield stress, the Ostwald–de Waele model was more appropriate for describing the flow behaviour of the samples.

The findings of our study indicated that the emulsifier application in the coconut emulsion (KSE) and almond emulsion (MSE) resulted in a more pseudoplastic behaviour with increasing consistency coefficient and decreasing flow behaviour index. OSE and OKSE showed an opposite pseudoplastic flow behaviour pattern: the consistency coefficient decreased when the emulsifier was applied. This might be caused by the different composition, the percentage of a raw material in the emulsions and the natural occurrence of fat in the raw materials used. It is known that coconut and almonds contain more fat than oats and rice. For this reason, lecithin as an amphiphilic emulsifier was used in rice cream recipe formulation, because rice had a minimal fat content (0.5 w.%). The lipophilic emulsifier (mono- and diglycerides of fatty acids) provided better properties for emulsions with a higher fat content. The more fat there is in the emulsion, the more effective the emulsifier is. This can be observed both from the measured flow parameters and viscoelastic curves.

Coconut emulsion provided a higher yield stress (*τ*_0_), higher consistency coefficient (*k*) and lower flow behaviour index (*n*), compared to other samples, which could be explained by the 53 vol.% content of grated coconut in the emulsion and 69 w.% of fat in raw material. It can be assumed that the grated coconut particles of a low bulk density had an effect on the resulting consistency. Therefore, a higher limit shear stress was necessary to achieve the flow state of the coconut cream, which was consistent with the findings for coconut creams’ rheological behaviour presented by Nimbkar et al. [13].

**Figure 1:**
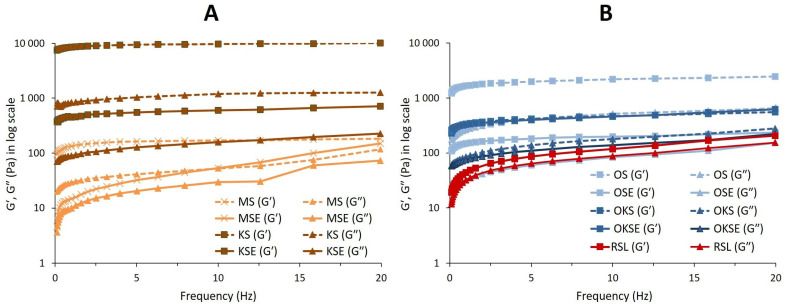
Viscoelastic moduli of plant-based creams with higher fat content (part (**A**)) and creams with a lower fat content (part (**B**)) in frequency range of 0.1–20 Hz. Samples without emulsifier are represented by dashed lines; samples with emulsifier are represented by solid lines. Data of elastic modulus *G*′ are depicted by cross symbols (×) or full squares (■); data of viscous modulus *G*″ are depicted by full triangles (▲).

**Figure 2:**
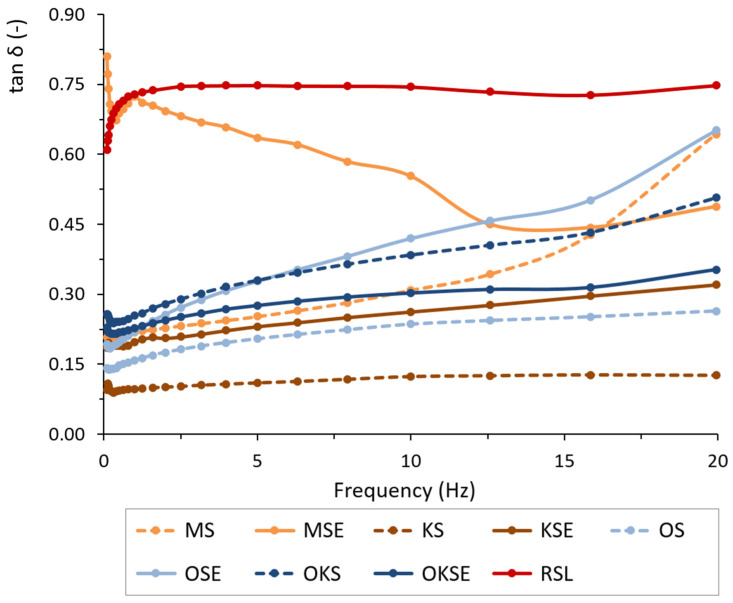
Phase angle *tan δ* of plant-based creams in the frequency range of 0.1–20 Hz. Samples without emulsifier are represented by dashed lines with data points; samples with emulsifier are represented by solid lines with data points.

To determine the viscoelastic properties of studied creams and to evaluate the storage modulus (*G*′), loss modulus (*G*″), and phase angle (*tan δ*), the frequency sweep was performed. The mechanical spectra (viscoelastic moduli as a function of frequency) obtained at the 25 °C and 0.1% strains are plotted in Figure 1. The predominance of the storage modulus over the loss modulus in the whole range of frequencies studied indicated that all creams exhibited a gel-like behaviour, probably related to an internal three-dimensional network structure [37]. The results showed two groups of plant-based creams with different viscoelastic behaviour, as can be seen in Figure 1. Oat–coconut creams (OKS, OKSE) exhibited viscoelastic properties different t other samples. For these cream types, the viscoelasticity was enhanced by the emulsifier application, as shown by lower values of phase angle (*tan δ*) determined for OKSE in the applied frequency range (Figure 2). We hypothesise that the mix composition of oatmeal and coconut particles (1:1 weight percentage ratio), with a higher volume percentage of coconut particles, caused a decrease in *tan δ*, i.e., the emulsifier slightly increased the viscoelasticity of OKSE.

Almond and coconut creams (MS, KS) behaved as emulsion materials whose viscoelasticity was optimisable by the emulsifier application (MSE, KSE). Coconut creams (KS, KSE) behaved as gel matrices characterised by a pronounced plateau region in the elastic part *G*′ of the complex modulus (Figure 1). In the plateau region, *G*′ was considerably higher than viscous part *G*″ of the modulus, thereby meeting solid-like gel characteristics [38,39,40]. Coconut cream KS showed the most intense viscoelastic behaviour, characterised by obtained lower values of *tan δ*, as can be seen in Figure 2. This behaviour may be related to the colloidal stabilization effect of coconut proteins and phospholipids [41], as well as to the low bulk density of coconut ingredient. It was also in correlation with a high stability index determined for KS (as discussed in Section 3.4), which might be an important factor contributing to the creams’ viscoelasticity. Following the emulsifier application, the KSE had viscoelastic moduli that were an order of magnitude lower than those observed without the emulsifier (KS). The addition of the emulsifier reduced the elastic modulus of both the coconut (KSE) and almond cream (MSE). This change was reflected in the increase in their loss (phase) angle, as illustrated in Figure 2. In both creams (KSE, MSE), the emulsifier and guar gum application simultaneously facilitated water absorption in the emulsion matrix, which enhanced the consistency of the creams, thereby reducing their viscoelasticity. The viscoelastic profiles of KSE and MSE creams agreed with their flow behaviour, indicating a more continuous gel-like structure. In the case of rice emulsion (RSL), the presence of lecithin and starch-based character of rice contributed to a lower viscoelasticity of RSL. Hence, a higher *tan δ* was observed (Figure 2), indicating a significant non-elastic component in RSL emulsion, compared to other cream types.

### 3.2. Texture Analysis of Plant-Based Creams

**Figure 3:**
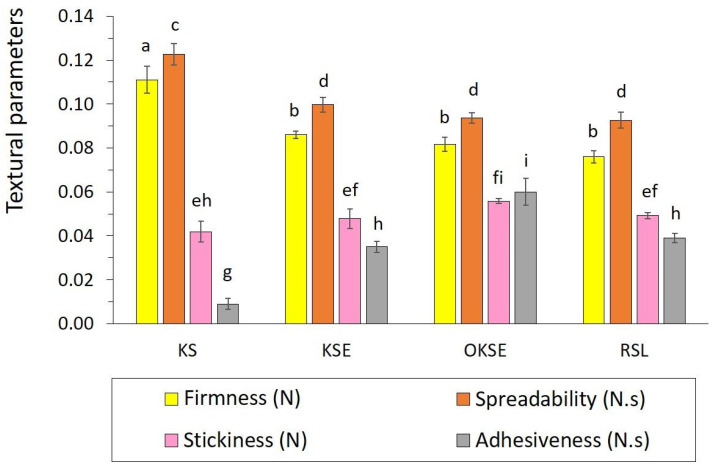
Texture profile analysis of plant-based creams measured by the 2 mm penetration test. Bar charts represent arithmetic means with error bars of standard deviation (*n* = 3). Different superscript letters above the bars indicate significantly different values (Tukey’s test, *p* ˂ 0.05). Samples not applicable to texture analysis are not involved in the graph.

Samples with the appropriate consistency of KS, KSE, OKSE, and RSL were applicable for texture analysis, as can be seen in Figure 3. Studied samples were comparable in textural attributes observed, i.e., firmness, spreadability, stickiness, and adhesiveness. As is evident, the addition of emulsifier had no statistically significant effect on firmness, spreadability, or stickiness; adhesiveness was slightly higher for OKSE. KS showed significantly higher values of firmness and spreadability and reduced adhesiveness, which could be related to higher elasticity of this sample, as discussed in Section 3.1. The addition of emulsifier (KSE) resulted in an increase in adhesiveness and reduction in firmness and spreadability. It might be explained by interactions between the emulsifier with proteins/saccharides by hydrophobic interactions, leading to the unfolding of proteins and the increasing affinity of the sample to the surface [42]. The results are in accordance with the fact that plant-based cream spreadability is inversely proportional to viscosity [43,44], indicated by the change in viscous/elastic component in the samples. Plant-based cream properties were affected by the ingredients’ composition, particularly oil/fat content [45], and the application of emulsifiers with a practical intention to optimise the texture profile of prepared products. Textural parameters of samples in the present study were comparable with the gel firmness determined by Gupta et al. [46] for plant-based yogurts.

### 3.3. Colour Analysis of Plant-Based Creams

The results of colour analysis are presented in Table 3. Based on the chromaticity coordinates, the creams can be described as light-yellow foodstuffs (characterised by relatively low +b* values) with a very slight green to red tint (low −a* or +a* values, respectively). As can be seen in Table 3, almond and coconut creams with emulsifier (KS, MS) and without emulsifier (MSE, KSE) exhibited relatively high lightness. On the other hand, oat cream (OS) and rice cream (RSL) were darker in colour compared to other samples. Nevertheless, it was found that the emulsifier application did not significantly affect the lightness of most creams. The same trend was observed for chroma C*, showing only minor differences between the creams of the same type. It is worth noting that the colour saturation of all samples under study, as indicated by C* data, was relatively weak, regardless of the cream type (Table 3). The data demonstrated no general relation between C* and L* parameters.

**Figure 4:**
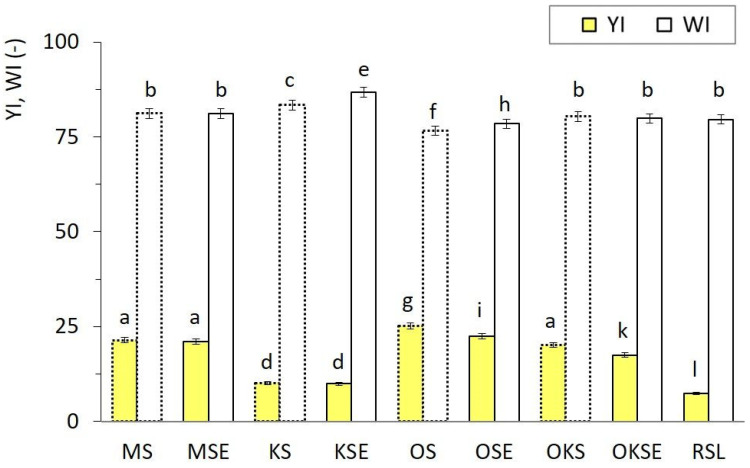
Yellowness index *YI* (yellow bars) and whiteness index *WI* (white bars) of plant-based creams without emulsifier (dotted lines) and with emulsifier (solid lines) measured in reflectance mode. Bar charts are presented as arithmetic means of three measurements with the error bars representing the standard deviations. Different superscript letters above the bars indicate significantly different values (Tukey’s test, *p* ˂ 0.05).

The values of hue angle h* observed in the range 80.8 to 87.0° indicated that the dominant spectral component of the creams was yellow. RSL in reflectance mode showed a less intense yellow hue shifted to the red tones (65.00 ± 0.11)°, which could be related to the presence of lecithin, as compared with other samples. The whiteness index *WI* and yellowness index *YI* were dependent on the cream type. However, the differences between the values of each index were relatively low (Figure 4). No general relationship was found between *WI* and *YI* parameters, although it was observed that OS and OSE samples with the lowest *WI* showed the highest *YI*, indicating relatively intense yellow tones of oat cream types. This fact was also confirmed by relatively high values of h* and C* (OS, OSE), as evident from Table 3. In contrast to this, the RSL sample characterised by the least intense chroma, lightness, and hue angle showed the lowest yellow tones *YI* (7.40 ± 0.15).

The colour profiles of plant-based creams can be compared with the data of relevant food products, such as spreadable cheeses and dairy alternatives. The colour parameters of dairy cream products were comparable with the results of our study, particularly in terms of the lightness and a* (redness to greenness) of the samples [31,34,35]. However, the versatile values of *YI* and *WI* can be related to the varying content of carotenoids and/or other pigments present in food ingredients used [47].

### 3.4. Colloidal Stability of Plant-Based Creams

**Figure 5:**
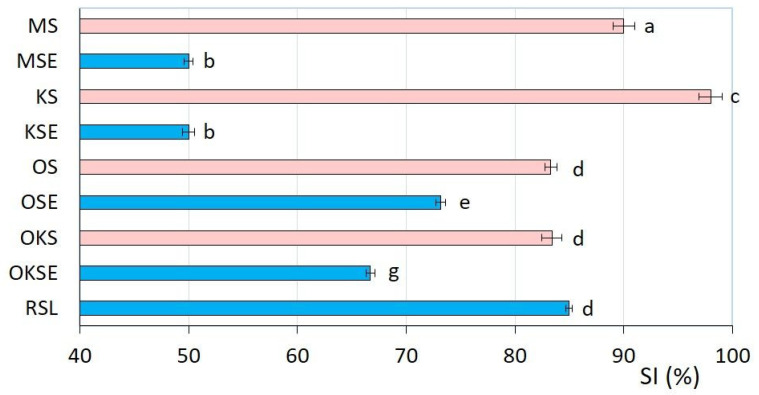
Emulsion stability index *SI* (%) of plant-based creams determined by centrifugation separation method (bar charts with error bars represent arithmetic mean of three measurements with the standard deviations). Samples without emulsifier are presented in red colour; samples with emulsifier are presented in blue colour. Different superscript letters next to the bars indicate significantly different values (Tukey’s test, *p* ˂ 0.05).

As is evident from Figure 5, relatively high emulsion stability (above 80%) was observed for MS, KS, OS, OKS, and RSL samples. The highest stability index (*SI*) determined for KS may be associated with the presence of dispersed coconut proteins and phospholipids in the cream structure. The main role might be attributed to cocosin, the most abundant storage protein of coconut, which is able to stabilise o/w emulsions by surface-active molecules; these molecules can absorb on the surface of oil droplets (at the oil–water interface) and prevent the aggregation of the oil phase via repulsive forces [41,48,49]. This emphasised the effect of proteins in stabilizing plant-based creams by creating a protective layer around the oil droplets, i.e., the effect of protein–lipid interactions [50]. However, the application of emulsifier can alter the dynamic of these interactions and thus the creams’ colloidal stability.

Oat cream demonstrated a slight decrease in *SI* after emulsifier addition (OSE). A significant decrease in emulsion stability was observed for almond and coconut creams with emulsifier (MSE, KSE), as shown in Figure 5. These results could be associated with the reduction in MSE and KSE viscoelasticity (i.e., with the softening of the creams’ structure, as discussed in Section 3.1). The reduction in creams’ *SI* may be aligned with the orogenic mechanism, characterised by the partial replacement of interface protein by the emulsifier used. The emulsifier, due to its higher surface activity, can displace the interface proteins and reduce the surface tension in the local domains of emulsion structure. This could potentially lead to a compression of protein network and thus to the reduction in the colloidal stability of the creams [51,52].

It was found that the stability index of samples under study was reduced by the mono- and diglycerides application. Contrastingly, rice cream with lecithin (RSL) yielded high emulsion stability (85.0 ± 0.3) %, thereby supporting previous studies suggesting the effective utilisation of lecithin in o/w emulsions [53,54,55]. Emulsifier application enhanced or reduced the colloidal stability of plant-based creams dependent on protein and fat content in substances used. The emulsifier amphiphilic nature reduces the surface tension and provides a steric barrier against the droplets coalescence [56,57]; the colloidal stability of plant-based creams can be effectively altered by emulsifier used.

## 4. Conclusions

This study provided advancement in understanding the rheological behaviour of various plant-based creams. The major finding was the synergistic effect of selected ingredients and emulsifier on the products’ rheological behaviour. Specifically, the formulated creams behaved as pseudoplastic liquids (*n* < 1), showing a reduction in shear viscosity after the application of fatty acids’ mono- and diglycerides. The increase in the consistency coefficient *k* and decrease in flow behaviour index *n* was observed for MSE and KSE after emulsifier application, indicating a formation of a stronger internal network structure. This finding suggested that MSE and KSE could be used in applications requiring a thicker consistency, such as cake fillings and toppings. The textural parameters of firmness, spreadability, stickiness, and adhesiveness were not significantly affected by the addition of emulsifier; the adhesiveness of OKSE was observed to be slightly higher.

The samples under study exhibited viscoelastic behaviour with an energy reversibility (*tan δ* ˂ 1) characteristic for gel-like products. The viscoelasticity of plant-based creams (except oat–coconut cream OKSE) was reduced after the emulsifier addition, represented by observed higher *tan δ*. OKSE was the only one cream type that showed increased viscoelasticity when emulsifier was applied. For RSL as a high-carbohydrate product with a minimal fat content, lecithin was applied. RSL viscoelastic properties were characterised by a significant non-elastic component compared to other samples. RSL could be utilised as food thickener in instant soups and convenience foods. From an economical point of view, it provided the relevant amount of cream using the lowest vol. % content of the basic ingredient (rice), compared to the other cream types. Optimised rheological properties could make the plant-based creams suitable in various culinary applications: they could serve as alternative products for people with lactose intolerance, providing a solution that is both health-conscious and tastes good. This highlights the potential of these creams to meet the needs of a growing consumer base seeking dairy alternatives.

Colour analysis revealed that the dominating colour spectral component of the creams was yellow, as the hue angle h* was ranging from 80.8 to 87.0°. Chroma C* values indicated that the colour purity of the samples was low and was not significantly changed by the emulsifier application. Plant-based creams could be used as visually appealing toppings for confectionery and baked goods, adding a colourful dimension without altering the products’ original colour significantly. The creams showed high colloidal stability, which decreased after the application of mono- and diglycerides of fatty acids. The stability index determined for RSL with lecithin was 85.0 ± 0.3%. The content of substances used and o/w ratio in plant-based emulsions played a basic role in the effectiveness of emulsifier applied. The alteration of creams’ stability, coupled with the ease of their preparation, makes plant-based creams potential candidates for commercial production as dairy cream alternatives. Future studies could investigate the potential health benefits of the creams, as well as their sensory acceptability among consumers.

## Figures and Tables

**Table 1:** Plant-based creams optimised recipe formulations calculated per 100 g of the basic ingredients, i.e., almonds, coconut, oatmeal, oatmeal–coconut at a 1:1 (*w*/*w*) ratio, and rice.

Sample (Abbreviation)	Ingredient Ratio (vol. %) ^a^	Water Content (mL)	Oil Content (mL)	o/w ^b^	Guar Gum (g)	Fine Rice Flour (g)	Salt (g)
**Creams without emulsifier**							
Almond cream (MS)	38.2	250	25	0.10	0.25	- ^c^	1.0
Coconut cream (KS)	53.0	250	25	0.10	0.50	50	1.0
Oat cream (OS)	46.0	250	25	0.10	0.50	-	1.0
Oat–coconut cream (OKS)	49.0	250	25	0.10	0.50	25	1.0
**Creams with mono- and diglycerides of fatty acids (0.75 g/100 g)**							
Almond cream (MSE)	38.2	250	25	0.10	0.50	-	1.0
Coconut cream (KSE)	53.0	250	25	0.10	0.50	50	1.0
Oat cream (OSE)	46.0	250	25	0.10	0.50	-	1.0
Oat–coconut cream (OKSE)	49.0	250	25	0.10	0.50	25	1.0
**Cream with lecithin (0.40 g/100 g)**							
Rice cream (RSL)	11.1	700	100	0.14	0.70	25	2.0

^a^ The value represents volume percentage of basic ingredient in the prepared sample. ^b^ o/w—oil/water ratio. ^c^ The hyphen means that the ingredient was not added to the recipe.

**Table 2:** Flow parameters of plant-based creams measured at 25 °C in the shear rate range of (0.1–100) s^−1^ and fitted by the rheological models of Herschel–Bulkley and Ostwald–de Waele.

Sample	Herschel–Bulkley Model				Ostwald–de Waele Model		
	*τ*_0_ (Pa)	*k* (Pa·s^n^)	*n*	*R* ^2^	*k* (Pa·s^n^)	*n*	*R* ^2^
MS	0.91 ± 0.04 **^ad^**	0.26 ± 0.02 **^a^**	0.75 ± 0.03 **^a^**	0.9948	1.30 ± 0.05 **^a^**	0.34 ± 0.01 **^a^**	0.9521
MSE	0.29 ± 0.01 **^a^**	0.57 ± 0.03 **^a^**	0.59 ± 0.02 **^b^**	0.9990	0.98 ± 0.03 **^a^**	0.43 ± 0.02 **^b^**	0.9802
KS	34.65 ± 1.68 **^b^**	1.71 ± 0.07 **^a^**	0.72 ± 0.02 **^ac^**	0.9553	2.08 ± 0.08 **^a^**	0.69 ± 0.03 **^c^**	0.9538
KSE	0.00 ± 0.00 **^a^**	47.39 ± 1.56 **^b^**	0.24 ± 0.01 **^d^**	0.9991	18.25 ± 0.57 **^b^**	0.45 ± 0.01 **^b^**	0.9858
OS	0.00 ± 0.00 **^a^**	204.90 ± 6.04 **^c^**	0.20 ± 0.01 **^d^**	0.9742	89.92 ± 2.14 **^c^**	0.35 ± 0.01 **^a^**	0.9915
OSE	6.77 ± 0.29 **^c^**	2.04 ± 0.08 **^a^**	0.67 ± 0.03 **^c^**	0.9992	9.95 ± 0.40 **^d^**	0.28 ± 0.01 **^d^**	0.9552
OKS	2.26 ± 0.11 **^d^**	15.41 ± 0.59 **^d^**	0.51 ± 0.02 **^e^**	1.0	18.20 ± 0.61 **^b^**	0.47 ± 0.02 **^be^**	0.9995
OKSE	0.00 ± 0.00 **^a^**	11.37 ± 0.41 **^d^**	0.45 ± 0.01 **^e^**	1.0	9.17 ± 0.34 **^d^**	0.51 ± 0.02 **^ef^**	0.9983
RSL	5.64 ± 0.25 **^c^**	10.21 ± 0.23 **^d^**	0.66 ± 0.03 **^bc^**	0.9974	16.34 ± 0.55 **^b^**	0.54 ± 0.02 **^f^**	0.9800

*τ*_0_—yield stress; *k*—consistency coefficient; *n*—flow behaviour index; *R*^2^—correlation coefficient. Results are presented as arithmetic mean ± standard deviation of three measurements. Mean values within a column followed by different superscript letters are significantly different (Tukey’s test, *p* ˂ 0.05).

**Table 3:** Colour analysis of plant-based creams characterised by L*, a*, b*, h*, and C* coordinates.

Sample	L*	a*	b*	h* (°)	C*
MS	86.40 ± 0.02 **^ab^**	1.56 ± 0.00 **^a^**	12.96 ± 0.03 **^ab^**	83.13 ± 0.01 **^ab^**	13.05 ± 0.02 **^ac^**
MSE	86.10 ± 0.01 **^ab^**	1.54 ± 0.00 **^a^**	12.71 ± 0.00 **^ab^**	83.09 ± 0.00 **^ab^**	12.80 ± 0.00 **^ac^**
KS	84.47 ± 0.14 **^ac^**	−0.97 ± 0.01 **^b^**	5.94 ± 0.03 **^c^**	80.76 ± 0.10 **^b^**	6.01 ± 0.03 **^b^**
KSE	88.27 ± 0.11 **^b^**	−0.85 ± 0.00 **^b^**	6.11 ± 0.00 **^c^**	82.08 ± 0.00 **^b^**	6.17 ± 0.00 **^b^**
OS	81.56 ± 0.01 **^de^**	1.26 ± 0.00 **^ac^**	14.35 ± 0.00 **^a^**	84.98 ± 0.00 **^c^**	14.40 ± 0.00 **^a^**
OSE	82.84 ± 0.02 **^cd^**	0.90 ± 0.01 **^cd^**	12.99 ± 0.00 **^ab^**	86.06 ± 0.02 **^cd^**	13.02 ± 0.01 **^ac^**
OKS	84.37 ± 0.00 **^c^**	0.62 ± 0.01 **^d^**	11.89 ± 0.00 **^b^**	87.04 ± 0.02 **^d^**	11.90 ± 0.01 **^c^**
OKSE	82.59 ± 0.21 **^cd^**	0.55 ± 0.02 **^d^**	10.10 ± 0.09 **^b^**	86.86 ± 0.11 **^d^**	10.12 ± 0.09 **^d^**
RSL	80.12 ± 0.00 **^e^**	−1.94 ± 0.01 **^e^**	4.15 ± 0.01 **^c^**	65.00 ± 0.11 **^e^**	4.58 ± 0.01 **^e^**

L*—lightness; a*—chromaticity on a green(−)-to-red(+) axis; b*—chromaticity on a blue(−)-to-yellow(+) axis; h*—hue angle; C*—colour saturation (chroma). Results are presented as arithmetic mean ± standard deviation of three measurements. Mean values within a column followed by different superscript letters are significantly different (Tukey’s test, *p* ˂ 0.05).

## Data Availability

The original contributions presented in the study are included in the article and the Supplementary Materials; further inquiries can be directed to the corresponding author.

## Supplementary Materials

The following supporting information can be downloaded at: https://www.mdpi.com/article/10.3390/foods13081225/s1. Table S1: Rheological data of plant-based creams.
